# The Role of Agriculture in the Dissemination of Class 1 Integrons, Antimicrobial Resistance, and Diversity of Their Gene Cassettes in Southern China

**DOI:** 10.3390/genes11091014

**Published:** 2020-08-28

**Authors:** Niyaz Ali, Yinfu Lin, Zhen Qing, Dan Xiao, Ahmad Ud Din, Izhar Ali, Tengxiang Lian, Baoshan Chen, Ronghui Wen

**Affiliations:** 1 State Key Laboratory for Conservation and Utilization of Subtropical Agro-Bio-Resources, College of Life Science and Technology, Guangxi University, Nanning 530004, China; 1708401009@st.gxu.edu.cn (N.A.); 1608403003@st.gxu.edu.cn (Y.L.); 1708401006@st.gxu.edu.cn (Z.Q.); 1808301057@st.gxu.edu.cn (D.X.); Izharali48@gmail.com (I.A.); chenyaoj@gxu.edu.cn (B.C.); 2 Drug Discovery Research Center, South West Medical University, Luzhou 646000, China; ahmadnwa@swmu.edu.cn; 3 State Key Laboratory for Conservation and Utilization of Subtropical Agro-Bio-Resources, South China Agricultural University, Guangzhou 510642, China; liantx@scau.edu.cn; 4 Guangxi Key Lab for Sugarcane Biology, College of Agriculture, Guangxi University, Nanning 530004, China

**Keywords:** integrons, gene cassettes, agriculture system, antimicrobial resistance

## Abstract

Integrons are hot spots for acquiring gene cassettes from the environment and play a major role in the bacterial evolution and dissemination of antimicrobial resistance (AMR), thus posing a serious threat. There are currently studies on integrons and antibiotic resistance genes; however, the presence and association of integrons in different agricultural crops and their subsequent dissemination and role in AMR have not been reported previously. This study examines the abundance of integrons, their gene cassette diversity in various crop soils, and their role in the dissemination of AMR in the southern region of China. Samples from different agri-crop soil, such as rice (R.S), sugarcane (S.S), citrus (C.S), banana (B.S), agricultural runoff (the point where the runoff of all sites meet (R.O)), and wild (non-agricultural) soil (W.S), were collected. Quantitative PCR was used to determine the abundance of integrons, and clone libraries were constructed to examine the gene cassette arrays. All the tested samples were found positive for Class-I (CL1) integrons and revealed a higher concentration and higher relative abundance of R.S than the others, with the least found at the W.S site. The W.S CL1 cassette arrays were found empty, and no putative conserved domains were found. The R.O was found to contain a high number of gene cassettes with various functions, while the smallest number of gene cassettes was found in the S.S among the crop soils. Most of the gene cassettes presented by the R.O were primarily shared with other sites, and the antibiotic-resistant genes were consistently observed to be dominant. The constructed clone libraries represented a diverse gene cassette array with 16% novel gene cassettes that play a vital role in pathogenesis, transportation, biosynthesis, and AMR. Most resistance-related gene cassettes were associated with the genes encoding resistance to quaternary ammonium compound (QAC) and aminoglycosides. This study highlights the significant differences in the abundance of integrons among various agricultural soils and offers deep insight into the pools of gene cassettes that play a key role in the dissemination of integrons and AMR.

## 1. Introduction

Bacterial genomes contain versatile gene accretion systems called integrons, which are commonly found in most gram-negative bacteria. Integrons are documented to be involved in the propagation and expression of genes in the bacterial community and play a major role in bacterial evolution, including antimicrobial resistance (AMR) development [1,2]. Integrons contain two common characteristics, a structural part and an array of gene cassettes (GCs). The 5’ conserved region (5’C.S) consists of an integron integrase (*intI*) gene, an integron-associated recombination site (attI), and an integron-associated promoter (Pc) site. This platform captures and expresses GCs that are non-replicative mobile elements in the bacterial genome. These GCs consist of one or more open reading frame (ORF) and recombination cassette, joining the attC site. A site-specific tyrosine recombinase enzyme called integron integrase is encoded by the *intI* gene, which has a specific activity of integration and excision of GCs. The expression of integrase genes can be induced by the accumulation of single-stranded DNA in a bacterial cell via conjugation, transformation, starvation, and/or exposure to antibiotics [3,4]. Integrons play a key role in bacterial evolution through the adaptation of GCs and the expression of their different ORFs along with their subsequent role in developing adaptive antibiotic resistance [5,6]. Integrons are classified into three broad groups based on the phylogeny of their respective integrase genes. The first group is found in *Proteobacteria* from freshwater and soil environments, including the clinically important Class 1 and Class 3 integrons [7,8]. The second group is found in gamma *Proteobacteria* from marine environments and consists of Class 2 integrons and integrons found on the plasmids of *Vibrio* [7,8,9]. The third group of integrons features those whose integrase genes are oriented in the reverse direction of the first and second groups of integron integrase genes and are found in the members of *Spirochetes, Plantomycetes, Cynaobacteria,* and *Chlorobi* spp., which are native to various environments [7,10]. Among them, Class 1 integrons have been found to play a key role in the development of antibiotic resistance [11,12]. Hundreds of GCs carried by integrons have demonstrated their role in resistance to different classes of antibiotics, such as amino glycosides, beta-lactams, chloramphenicol, trimethoprim, and streptothricin [12,13]. 

Moreover, gene cassettes are widely dispersed in diverse environments, and different GCs are often expressed by the same bacterial species [14,15]. The functions of the predicted proteins of the ORFs within GCs are wide-ranging. In addition to antibiotic resistance, they are involved in virulence and secondary metabolism, which are more specific to these species [3,11]. Most studies on integrons and their metagenomic analyses demonstrated that most GCs are novel [16,17]. To investigate GCs in different environments, the PCR-based amplification of GCs is commonly used following a metagenomic analysis using genomic DNA as a template [18,19]. Furthermore, many studies proved the dispersion of integrons in a wide range of environments, such as sediments, rivers, oceans, soils, plant surfaces, the rhizosphere, bio-films, hot springs, and Antarctic soils [9]. Furthermore, the presence of antibiotics in agriculture water [20] may affect the soil microbial community [21], as well as instigate antimicrobial resistance in soil microbes [22]. On the other hand, the presence of various soil contaminants, soil compositions, and microbial biomasses may influence the soil microbiome and enhance the dissemination of antibiotic resistance genes (ARGs) [23,24].

Manure is commonly used as an organic fertilizer to upgrade the fertility of agricultural soils. The induction of manure to the soil introduces vast bacterial communities that carry antibiotic resistance genes (ARGs) present on mobile genetic elements (MGEs) [25,26]. These ARGs are transferred into the soil’s bacterial communities through a process called horizontal gene transfer [27]. The application of manure may result in the enrichment of bacterial taxa, including human pathogens [28]. On the other hand, the soil’s natural reservoirs present their own sets of known and unknown ARGs [29]. Due to the selective pressure imposed by the application of antibiotic agents (ABAs) to the soil, the resilience of soil bacteria against manure application is decreased in the presence of ABA [28]. The wastewater, treated water, and lake water all contain ARGs [30]. Thus, the water used for irrigation purposes from these sites poses a risk of transmitting antibiotic resistance ABR and ARGs [31]. Organic and inorganic fertilization impacts the abundance of ABR and integrons in the soil environment by introducing resistant bacteria [32,33]. 

The dissemination of integrons is very common in most of the environment, and anthropogenic inputs play a vital role in this phenomenon [34]. The agricultural system covers a vast environment and requires a deep insight to investigate the representation of CL1 integrons and their GCs, which reside in these crops’ environments. Thus far, there have been no studies on various agri-crops and their impacts on both integrons and the gene cassette pools at these sites, especially on the crop soil environment and the effluent discharge from these environments. The current study investigates integron dissemination and GCs in different crop systems and establishes their contributions to the rise of antimicrobial resistance.

## 2. Materials and Methods

### 2.1. Samples Collection

Soil samples were collected from a typical farming area (between longitude 107°58′–107°90′ and latitude 22°47′–22°55′) located at Fusui county, which is a typical karst region in southern China whose main crops include sugarcane, rice, bananas, and citrus. Soil samples were collected from each of these agriculture fields, considering each crop field as a separate site for sample collection. The soils of different fields, including rice soil (R.S), citrus fields (C.S), sugarcane fields (S.S), banana fields (B.S), wild soil (non-agricultural sites where the anthropogenic input is very limited, W.S), and agricultural runoff (the point where the runoff of all sites meet, R.O), were collected. The collected samples were then preserved at −80 °C for further use. The soil properties including pH, organic matter (OM), available phosphorous (AP), available potassium (AK), total potassium (TK), total phosphorous (TP), total nitrogen (TN), available nitrogen (AN), total carbon (TC) and C:N ratio were measured as previously described by Ali et. al. [35] and the values are presented in Table 1. 

### 2.2. DNA Extraction

DNA was extracted using the soil DNA extraction kit according to the manufacturer’s instruction (QIAGEN, Korea Ltd., Seoul, Korea), and the concentration of DNA in each sample was quantified using Nano drop (model one C). The extracted DNA samples were preserved at −80 °C for the follow-up experiments.

### 2.3. CLASS 1 Integrons Detection and 16SrRNA

To amplify the Class 1 integron, forward (5′-ATCATCGTCGTAGAGACGTCGG-3′) and reverse primers (5′-GTCAAGGTTCTGGACCAGTTGC-3′) were used as described previously by Rosser et al. [36]. The primer pair for 16SrRNA (forward 5’-CGGTGAATACGTTCYCGG-3’ and reverse primer 5’-GGTACCTTGTTACGACTT-3) was used as described by Gaze et al. [37]. The PCR reactions were conducted in a reaction volume of 50 µL, and the amplified fragments were separated using gel electrophoresis for visualization. The different bands were cut from the gel and extracted using a gel extraction kit (Vazyme, Nanjing, China) according to the manufacturer’s instructions. The DNA fragments were cloned into a blunt cloning vector using a TA/Blunt-Zero Cloning Kit (Vazyme, Nanjing, China), according to the manufacturer’s instructions, and accordingly transferred into *E. coli* DH_5α_ competent cells. These cells were then cultured overnight on LA medium containing ampicillin at a concentration of 100 µg/mL. The positive colonies were selected after overnight incubation, and the plasmids were extracted from the culture using a plasmid extraction kit (Vazyme, Nanjing, China). These recombinant plasmids were Sanger sequenced via a universal sequencing primer at AuGct Biotechnology Co. Ltd. (Beijing, China).

### 2.4. The Gene Cassette Libraries

To amplify the variable regions of Class 1 integrons, primer pairs were used [38] in a reaction mixture of 50 μL containing 20 ng of DNA, 25 μL 2 × premix Takara Ex Tag (Takara, Kusatsu, Japan), and 2.5 μL of both primers. The PCR conditions were optimized as described previously [38,39]. Triplicates were amplified from each DNA sample, and the amplicons were pooled and purified with a Vazyme gel purification kit (Vazyme, Nanjing, China). The PCR purified products were then quantified and ligated into the M13 vector and transferred into the competent cells of *E. coli* DH_5α_ following the manufacturer’s instructions (Vazyme, Nanjing, China). From each sample, 80 clones were selected randomly and sequenced after validation using a gene cassette size of 153 bp via M13 reverse and forward primers. To avoid ambiguous bases, the sequences were confirmed after assembling the primer sequences, and the GCs were further analyzed when the sequences contained the two primer pairs (3′C.S and 5′C.S). The sequences were further annotated for nucleotide blast with BLASTN and BLASTX for protein [40]. The sequences that were annotated as hypothetical or had no hits in NCBI were further authenticated using the NCBI ORF finder (https://www.ncbi.nlm.nih.gov/orffifinder/).

### 2.5. Quantitative PCR (q-PCR)

To quantify the abundance of Class 1 integron integrase genes and 16s rRNA genes, qPCR was conducted for all these samples, as reported by Barrud et al. [41]. SYBR^®^ Premix Ex Taq™ II (Takara, Kusatsu, Japan) was used to conduct the qPCR using a Roche real-time PCR system. The conditions of amplifications were set as previously described [38,42], and the sample amplifications were carried out in triplicate. The standard curve was formed using serial dilution of the plasmids carrying the relevant gene fragments. The relative abundance of integron integrase genes was calculated by dividing the 16s rRNA gene copy number by the copy number of the integrase gene.

### 2.6. Statistical Analysis

Statistix 8.1 (Analytical Software, 2105 Miller Landing Rd, Tallahassee, FL, USA) was used for the data analysis, and the figures were plotted using the Sigma Plot 12.5 (Systat Software, Inc. 1735 Technology Drive, Ste 430, San Jose, CA, USA) software and Origin 9.1 (OriginLab Corporatio, One Roundhouse Plaza, Suite 303, Northampton, MA, USA). The means of the replicates were compared using a least significant difference test with a 0.05 probability level. The shared GCs between all samples were picked, and the sharing network was visualized using Gephi (V0.8.1) [43]. All the sequences were submitted to NCBI with accession numbers from MT499916 to MT500005.

## 3. Results

### 3.1. Presence of Integrons and Abundance of CL1 Integron and 16srRNA

Class (CL) 1 integron integrase genes were detected in all samples, and the 16s rRNA gene was amplified in all the collected samples. The CL2 and CL3 integrons were rarely detected in the agricultural effluent (R.O) and rice field samples (R.S), while the wild soil (non-agricultural soil) (W.S), sugarcane (S.S), banana (B.S), and citrus (C.S) soils were found to be negative for CL2 and CL3 integrons. CL2 and CL3 integrons are usually found in clinical settings and sometimes at wastewater treatment plants, which mostly contain human microbiota.

**Figure 1:**
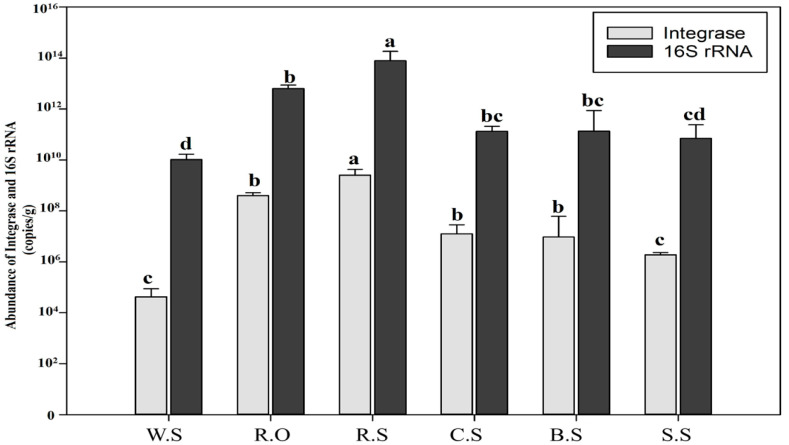
Concentration/copy number of the CL1 integron integrase gene and the 16S rRNA gene in each sample. The off-white bar indicates the concentration/copy number per gram of soil of the integrase gene in each soil sample, while the black bar indicates the 16Sr RNA copy number per gram of soil in each sample. Differences in the copy numbers of each sample group were evaluated using the Least Significant Difference (LSD). Different letters (a, b, c, d) above the column indicate statistical significance at *p* values < 0.05. W.S—wild site, R.O—agricultural runoff site, R.S—rice field site, C.S—citrus field site, B.S—banana site, S.S—sugarcane site. The wild soil sample contained the lowest concentration/fewest copy numbers of both integrase and 16S rRNA, while the highest copy numbers of both genes were found in the R.S samples followed by R.O, C.S, B.S, and S.S.

To determine the abundance/concentrations of integrons and 16s rRNA genes, qPCR was performed. The results showed that the abundance of CL1 integrons among all the samples was the highest in R.S, followed by R.O, while the fewest copies were observed in W.S (Figure 1). The 16s rRNA copy number was higher in the rice fields compared to the other samples, and the smallest number was found in the W.S (*p* < 0.05), which suggests that the high copy number of integrons in the other samples was due to the anthropogenic input. The other factors involved in the increased copy numbers in R.S include the introduction of manure and other fertilizers into the bulk amount of the fields, which were almost negative in the wild soil.

### 3.2. Class 1 Integron Gene Cassette Pool

**Figure 2:**
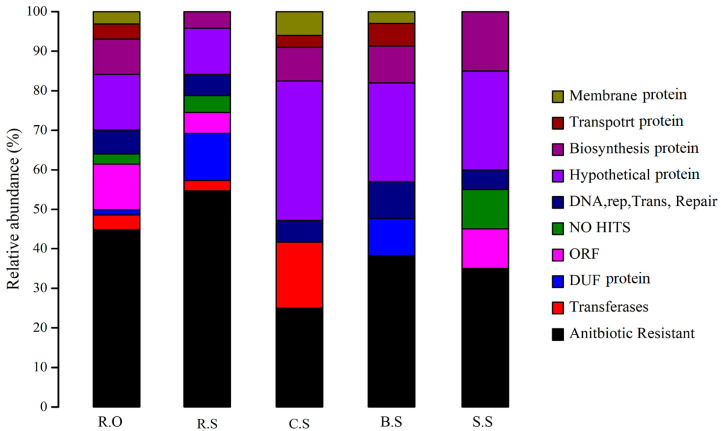
The relative abundance of different GCs transcribed into various proteins with various functions are represented by different colors from the bottom to the top of the bars. The distances covered by the various colors represent the relative abundance in that specific site. R.O—agriculture runoff site, R.S—rice field site, C.S—citrus field site, B.S-banana site, S.S—sugarcane site. A bar with more colors shows that the site is more diverse. In this figure, the R.O site is more diverse, while the S.S site has the fewest variants.

To further analyze the integrons and their GCs, a library of nearly 500 clones was built. About 60% of the GCs were found to be empty, carrying no putative sequences. In the wild soil samples, more than 100 clones were generated. None of the clones showed any putative proteins, which clearly indicates that the integrons were mostly active during periods of anthropogenic or environmental stress and that bacteria exchanged the GCs under stress conditions. Overall, about 12% of the GCs were annotated as novel and showed no hits in a NCBI Blast. The most diverse GCs were found in the effluent soil, and about 42 different GCs were identified while assembling the sequences, resulting in about 22 different proteins being transcribed. The fewest variant GCs were found in the sugarcane soil, which shared about six types of dissimilar GCs. The most abundant GCs were quaternary ammonium compound (QAC) resistance genes, which were prevalent in all samples. Figure 2 highlights the different GCs in these environments

### 3.3. Shared Gene Cassette Network

**Figure 3:**
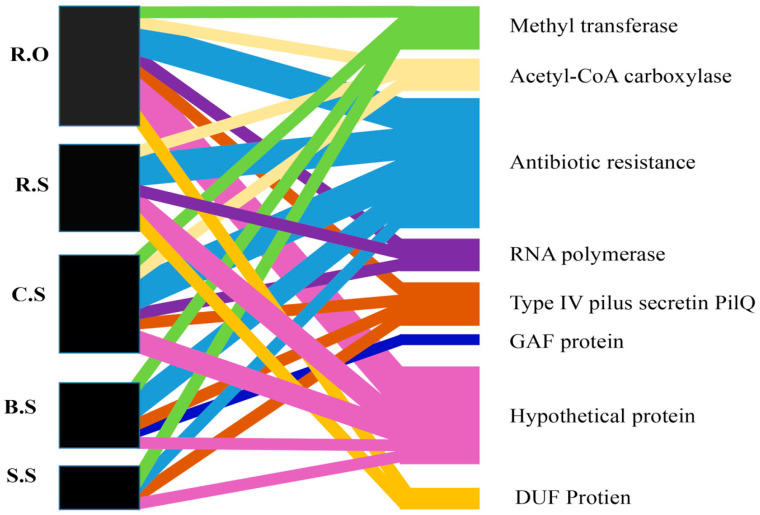
The shared gene cassette networks among the sites. The black box on the left side shows the sites, and the box’s horizontal width shows the different proteins in that site. The various colors on the right side of the figure represent the different gene cassette proteins. The crossed lines of various colors between the two blocks show the presence of a specific protein, and the width of the line shows that protein’s concentration in that specific site. R.O—agriculture runoff site, R.S—rice field site, C.S—citrus field site, S.S—sugarcane field site, B.S—banana field site. The line represents the association between various sites, while the different colors represent the various GCs.

A shared gene network was also drawn to represent the GCs shared among these environments. A total of eight GCs were shared by these sites. Most of the GCs were shared by the R.O and C.S sites. Among the shared GCs, the most common were the ones harboring various types of aminoglycoside resistant genes. The same method was followed for the rest of the GCs. The most relevant GCs were merged and are represented in Figure 3. The other gene cassette shared by all sites involves a hypothetical protein. The results show that these GCs flow from different sites and accumulate at the R.O site. 

### 3.4. Unknown Gene Cassette Pool

**Figure 4:**
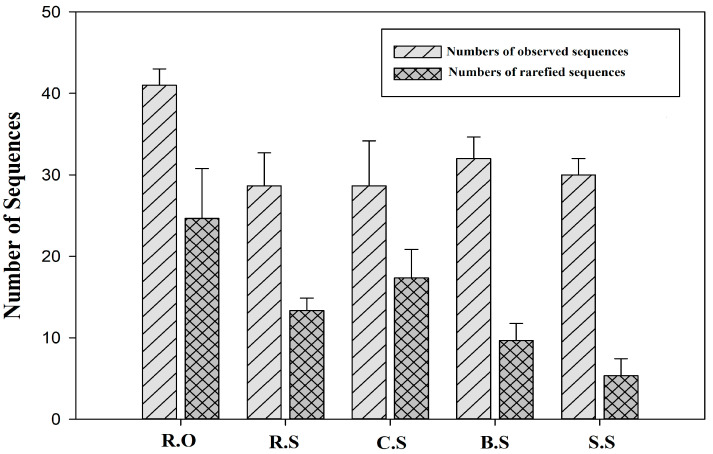
Number of observed and rarefied sequences (gene cassettes in each sample. R.O—agriculture effluent site, R.S—rice field site, C.S—citrus field site, B.S—banana site, S.S—sugarcane site. Each bar on the left indicates the average observed sequences, while the bars on the right indicate the rarefied sequences based on the clone libraries. Most rarefied sequences were higher for the R.O site, while the lowest numbers were found for the S.S site.

**Figure 5:**
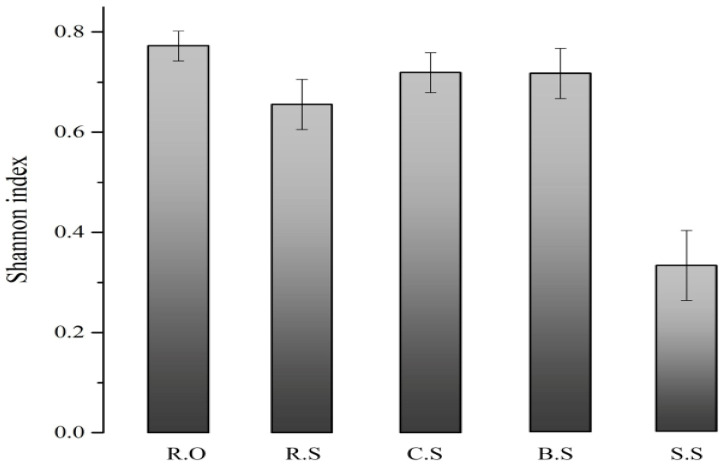
Diversity of GCs in Class 1 integrons in each sample. R.O—agriculture effluent site, R.S—rice field site, C.S—citrus field site, B.S—banana site, S.S—sugarcane site. The bars from left to right show the samples sites. From left to right, the most diverse site in terms of GCs was R.O, and the least diverse was S.S.

We also found some GCs related to stress, mobility, cell synthesis, chemotaxis, transport, and DNA repair. Interestingly, we found 1 kb GCs, which represent a particular type IV secretary conjugative DNA system that mediates interbacterial conjugative DNA transfer and the transfer of its DNA into eukaryotic cells such as *Agrobacteruim tumeficians.* Another 1.2 kb gene cassette was identified to carry the proteins of an ATP-binding cassette (ABC) transport system, which is an important part of the periplasmic transport system. Another gene cassette that transcribes the glycosyl hydrolase family 20 (GH20) catalytic domains of N-acetyl-beta-D-glucosaminidase enzymes, which is novel among the GCs of integrons. Moreover, this family features several functional forms of cell synthesis in pathogenesis. Figure 4 represents the observed and rarefied sequences. The analysis shows that the extensive sequencing of the GCs still did not identify all GCs in the sites. The Shannon index analysis shows that the diversity among the replicates is variable, which demonstrates that there are more diverse gene cassette pools among the various sites (Figure 5).

**Figure 6:**
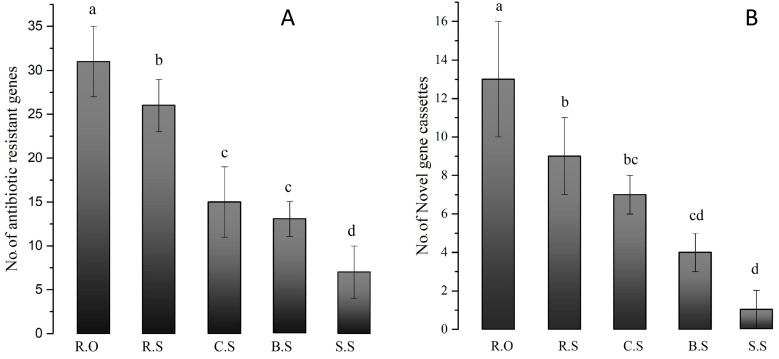
Numbers of antibiotic resistance genes (**A**) and novel GCs (**B**) observed in each sample. R.O—agriculture effluent site, R.S—rice field site, C.S—citrus field site, B.S—banana site, S.S—sugarcane sites. Different letters indicate different significant differences, while the error bars show the standard errors. Different letters (a, b, c, d) above the column indicate statistical significance at *p* values < 0.05. The highest number of antibiotic resistance GCs and novel GCs was found for the R.O site, and the lowest numbers were found for the S.S site.

Another gene cassette was found, which harbored the co-enzyme F430, which catalyzed the reduction of methyl co-enzyme M to methane. Mostly anaerobic bacteria contain this enzyme, but there were no homologies in the database, which indicates the novelty of these sequences found in the integron gene cassette array (Figure 6B). Other GCs that harbor an enzyme called topoisomerase-primase are involved in the DNA repair system. We also found some transcription regulator GCs that play a role in transcription.

### 3.5. Various Antibiotic Resistance GCs

Different antibiotic resistance GCs found in this study conferred resistance to amino glycosides, streptomycin, and penicillin. Resistant genes such as *aadA1 aadA2, aacA3, dfrA,* and *QacG* were frequently found in the agriculture effluent (*p* < 0.054), rice field, and soil samples. In contrast, aminogylcoside was found frequently in all samples (Figure 6A), except for the wild soil samples. Moreover, all cassettes from the wild soil samples were empty. Antibiotic resistance genes were frequently found in each sample, as described in Table 2.

## 4. Discussion

In this study, Class CL1 integrons were detected in soil samples from different agricultural systems, and the relative concentrations of 16Sr RNA were found to be highest in the rice field soil samples (R.S). In contrast, the lowest concentrations were found in the wild soil (W.S) samples. The incidence of CL1 integrons in the sugarcane field soil (S.S), banana field soil (B.S), and citrus field soil samples (C.S) was found to be lower than that in the agriculture effluent soil (R.O) and R.S. The net concentration of CL1 integrons was slightly lower than that previously reported in poultry waste or wastewater treatment plants but higher than that in freshwater sediments [38,39,44,45]. On the other hand, CL2 and CL3 integrons were barely detected (only found in R.S), whereas the agricultural runoff (R.O) and the other samples were found to be negative for these integrons. CL1 integrons were the most prevalent in the environment and are considered the dominant source for acquiring and spreading GCs [11,39]. Reports are limited on the detection of CL2 and CL3 integrons in soil environments [46,47], though their presence was observed in *E. coli* isolates of animal and human fecal samples [39,46,47]. Their occurrence was also observed in low concentrations in wastewater environments [38]. CL2 and CL3 have limited functions compared to CL1 integrons, and their evolutionary history explains this lower abundance [12]. The integrase associated with CL2 and CL3 integrons often exists in an inactivated form and the integrons then become unable to acquire the external GCs from the environment into their arrays [11,12,39]. Water is considered the main source of agricultural contamination as it carries CL1 integrons and GCs and is considered the most obvious reason for the dissemination of integrons in the agricultural environment. The amendment of struvite, poultry waste, and manure and sludge increased the abundance of CL1 integrons and the gene cassette arrays in the bacterial communities in the soil [48,49,50]. Organic and inorganic fertilizers are considered to be sources of integrons and might have affected the concentration of CL1 integrons in our study. The increased incidence of CL1 in the rice field might be associated with the comparatively higher use of fertilizers in that soil [32]. Recently, struvite and biochar applied to the field have been reported to play a role in the abundance of CL1 integrons and GCs, particularly in a rhizosphere environment [39].

GCs are a substantial element of genomic diversity and play an important role in the genomic diversity of bacterial evolution [11,39]. The gene cassette analysis of our study revealed that many GCs have no homology in the database or with any other conserved hypothetical proteins. These findings are consistent with those of other studies related to environmental investigations of integron GCs and their subsequent integration and transfer to microbial communities [17,32]. Interestingly, we found a 1kb gene cassette, which is a particular type IV secretary conjugative DNA system that mediates interbacterial conjugative DNA transfer and trans kingdom protein transfer into eukaryotic host cells in the process of pathogenesis. The sole bacterium that carries this type of gene is *Agrobacteruim tumefaciens* [51,52]. This gene also facilitates the adaptation of the organism to environmental stress and plays a basic role in the spread of antibiotic resistance among communities [53]. This observation further demonstrates that integron GCs play a vital role in the genetic evolution of bacteria through the acquisition of genes from other species [53,54]. Such areas are thus hotspots of antibiotic resistance evolution in bacteria and the reason for the horizontal gene transfer among them [32,39]. In this study, all the agricultural environments contained CL1 integrons and carried different types of antibiotic-resistant GCs. In contrast, the frequency of antibiotic resistance GCs was higher in the R.O sediments followed by R.S, C.S, S.S, and B.S. However, this frequency was still lower than that in hospital waste and wastewater treatment plants [38,45,55].

We found two novel GCs with sizes of 1.2 kb and 1 kb; the former belongs to the ABC transport system (specifically to the ModA system of the periplasm), while the latter is a twin arginine-targeting protein translocase TatC protein. TatC is a component of the twin-argentine transport system and is highly conserved. Arginine transport is required to transport the folded proteins across the inner membrane [53]. At the same time, defects in this component lead to defects in colonization [56]. Beta-N-acetyl hexosaminidases of glycosyl hydrolase family 20 (GH20) GCs were also found in the present study. This family is commonly found in microorganisms, animals, and plants that are involved in physiological and pathological processes such as cell structure, energy, defense, inflammatory signaling, and lysosomal storage [57]. We also found another gene cassette that was transcribed into the co-enzyme F430 synthase, which is a prosthetic group of methyl co-enzyme M reductase that catalyzes the reduction of methyl co-enzyme M to methane [58]. We also observed other novel GCs related to DNA synthesis and transcription regulators that translate the enzyme’s topoisomerase-primase and ring finger enzyme. Previous studies showed that these regulators have a high number of GCs related to biosynthesis and represent some GCs with the functions of chemotaxis, DNA repair, and regulation [38,39,45].

Most of all GCs were found to be associated with resistance to amino glycosides and streptomycin. These findings are consistent with the previous observations of environmental GCs from wastewater treatment plants, hospital water waste, struvite application [38,39], poultry litter [45,59], river water, and sediments [45]. Selective pressure from the environment may be responsible for the prevalence of antibiotic resistance GCs, and the co-selection of antibiotics at these sites along with heavy metals could be other factors facilitating the rise in the development of antibiotic resistance [60].

Different genes were identified in these samples, including *aadA1*, *aadA2*, *aacA3*, and *dfrA*. This result is in line with the conclusions of previously reported studies [45]. The excessive use of biocides for cleaning and washing purposes in hospitals, kitchens, and farms in the form of pesticides could be the main reason for these developments. This concern has been raised by several scientists, who suggested that the use of biocides in general practice, industrial settings, and agricultural farming is a contributing factor to the rise in antibiotic resistance bacterial strains [61]. Organic and inorganic fertilization impact the abundance of AMR and integrons in soil environments and can also introduce resistant bacteria [32,33,34]. 

Plant pathogenic bacteria can acquire resistance from soil and environmental bacteria, thereby making these plant bacteria more lethal to plants, as the roots can acquire soil bacteria containing GCs and then facilitate the migration of these GCs from the soil to the roots and, finally, to the phyllosphere. Soil resistant bacteria can enter the plant tissue through stomatal openings or mechanical injuries to the roots and can then be transported within the plant’s physiological system and colonize the leaves [33,39]. The differences between the GCs of the studied sites are consistent with those of previous studies. The gene cassette pools here were different from those with other geographical distributions, possibly due to the presence of different anthropogenic and natural inputs [16,22]. On the other hand, the presence of various soil contaminants, soil compositions, and types of microbial biomass may influence the soil microbiome and enhance the dissemination of ARGs [23,24]. Our study showed that the GCs are distinct from those in previously reported studies. Moreover, various sites have a distinct pool of GCs, and these various microbial communities may drive the proliferation and development of GCs in integrons [11]. For bacterial adaptation and evolution, GCs are the most significant components that interact with this specific environment [53,62].

## 5. Conclusions

Bacterial evolution is greatly advanced by integrons and between integrons. Class-I integrons play a core role in the dissemination of different genes across the ecosystem. The present study investigated the role of agriculture in the dissemination of integrons and determined their role in the development of antimicrobial resistance. This study highlights the increased concentration of integrons and the undiscovered pool of GCs. The incidence of antimicrobial GCs in all the investigated samples highlights the role of agriculture in the transmission of antimicrobial resistance. The presence of GCs representing the family glucosyl hydrolase further highlights the dispersal of important pathological genes in the agricultural system. The shared GCs between the effluent and crop soil environments, indicates that these s flow from soil environments to effluent environments, the latter of which often lead to streams and rivers, thereby playing a significant role in the dissemination of integrons and their cassette arrays into water bodies. In return, this process has an impact on both human and non-human populations, including plants. Continuous monitoring of Class-I integrons in different agricultural systems will further contribute to the current knowledge of bacterial evolution and aid in designing relevant reclamation strategies.

## Figures and Tables

**Table 1:** Properties of the different sites.

Properties	W.S	R.O	R.S	C.S	B.S	S.S
pH (water)	7.33	8.62	6.81	4.44	6.74	5.04
OM (g/kg)	13.57	17.53	47.16	36.18	53.57	15.89
AP (mg/kg)	5.23	60.37	31.00	278.3	66.33	8.02
AK (mg/kg)	22.07	68.17	132.76	278.6	347.39	39.53
TK (g/kg)	2.14	1.97	1.59	1.99	3.67	2.65
TP (g/kg)	0.78	0.74	0.92	1.19	2.50	0.64
TN (g/kg)	1.16	0.68	2.13	1.22	2.49	0.69
AN (mg/kg)	56.82	47.69	172.88	108.6	102.2	60.07
Total C (g/kg)	7.87	10.17	27.36	21.0	31.07	9.22
C:N ratio	6.81	15.02	12.87	17.27	12.47	13.31

Note: OM—organic matter, AP—available phosphorous, AK—available potassium, TK—total potassium, TP—total phosphorous, TN—total nitrogen, AN—available nitrogen, TC—total carbon and C:N ratio—carbon to nitrogen ratio, W.S—wild site, R.O—agricultural runoff site, R.S—rice field site, C.S—citrus field site, B.S—banana site, S.S—sugarcane site.

**Table 2:** Clone numbers of the antibiotic resistance GCs detected in all samples.

Gene family	Antibiotic Resistance Associated Gene	R.O	R.S	C.S	B.S	S.S
Aminoglycoside	*aadA1 gene*	3	3	4	1	1
Aminoglycoside	*aadA2*	1	1	0	0	2
Aminoglycoside	*glycoside hydrolase*	1	0	1	0	1
Aminoglycoside	*(aacA3)*	1	0	0	0	0
Quaternary ammonium resistance protein	*QacG*	3	1	6	1	0
Dehydrofolate	*DfrA*	2	0	0	0	0
Streptomycin	*Streptomycin 3′-O-adenylyltransferase*	2	2	4	1	1

Note: The first column shows the family of the gene, and the second row presents the associated gene, while the digit represents the number of GCs from that site. R.O—agriculture effluent site, R.S—rice field site, C.S—citrus field site, B.S—banana site, S.S—sugarcane site. The R.O sites present a diverse group of antibiotic resistance genes, while the fewest and least diverse antibiotic resistance genes are present in the S.S site.
